# Glutathione biosynthesis and metabolism in *Plasmodium falciparum*

**DOI:** 10.1186/1475-2875-9-S2-P37

**Published:** 2010-10-20

**Authors:** Eva-Maria Patzewitz, Sylke Müller

**Affiliations:** 1 Research Institute for Infection, Immunity & Inflammation and Wellcome Centre for Molecular Parasitology, School of Life Sciences, University of Glasgow, Glasgow, UK

## 

Intraerythrocytic *Plasmodium falciparum* live in an environment of increased oxidative stress primarily due to their metabolism of host cell haemoglobin. Therefore the parasites depend on functional and effective antioxidant systems. They possesses a glutathione redox system consisting of the tripeptide glutathione (GSH), the enzymes required for glutathione biosynthesis γ-glutamylcysteine synthetase (γGCS) and glutathione synthetase (GS), as well as a glutathione reductase, glutaredoxins I and II and a single glutathione-S-transferase.

As opposed to the murine malaria species *P. berghei* we show here that *P. falciparum* relies on a functional GSH biosynthesis during blood stage development with γGCS and GS being critical for parasite survival. Our data strongly suggest that there are significant metabolic differences between the human and murine malaria species that must be considered when evaluating and identifying potential drug targets. We further show that the importance for GSH biosynthesis in *P. falciparum* is attributable to the rapid efflux of glutathione from the infected red cell and the parasite's inability to scavenge sufficient amounts of the tripeptide from its environment to compensate this constant loss of GSH. The essential role for maintaining adequate intracellular GSH levels during blood stage development is further corroborated by the fact that inhibition of γGCS with the specific inhibitor L-buthionine sulphoximine causes a rapid decrease in cellular glutathione levels in the parasite and has a plasmodicidal effect. Over-expression of γGCS decreases the susceptibility to L-buthionine sulphoximine and results in a decrease in glutathione reductase levels. This presumably leads to an increased efflux of glutathione disulphide resulting in no apparent changes in intracellular GSH levels between wild-type and γGCS over-expressing parasites. These data indicate that the parasite's glutathione metabolism is tightly regulated by biosynthesis, reduction and efflux.

